# Characteristics and main controlling factors for the development of shale micro pore-fracture in Tiemulike Formation, Yining Basin

**DOI:** 10.1038/s41598-024-81804-1

**Published:** 2024-12-05

**Authors:** Yangwei Feng, Yan Ren, Wei Sun, Ting Jiang

**Affiliations:** 1 School of Geography and Tourism, Nanyang Normal University, Nanyang, 473061 China; 2 State Key Laboratory of Continental Dynamics, Northwest University, Xi’an, 710069 China; 3 Xi’an Center of Geological Survey, China Geological Survey, Xi’an, 710054 China

**Keywords:** Shale gas, Micro pore, Controlling factors, Thermal evolution, Brittle minerals, Geology, Petrology, Sedimentology

## Abstract

Research on the type, size, structure, and other characteristics of shale micro pore-fracture and their genesis is one of the core index for Shale gas study. Based on systematically collected shale samples from outcrop profiles and well cores, the experiments of thin-section observation, scanning electron microscopy, energy-dispersive X-ray spectroscopy, whole-rock analysis, rock–eval pyrolysis and basin simulation analysis were performed to study the micro pore-fracture characteristics and its main controlling factors for the development of shale pores in Tiemulike Formation in Yining Basin. The results show that four types of micro pore-fractures were identified: organic hydrocarbon-generating micro pores, granular dissolved micro pores, intergranular micro pores, and micro-fracture. The development of micro pores in shales is influenced by the internal material composition of the shale reservoir and external temperature conditions. The high organic carbon content with a high degree of thermal evolution led to the development of numerous micro pores, and the main micro pores were produced by shrinking the hydrocarbon generation volume due to the thermal evolution of organic matter. The development of micro-fractures was found to be favoured by the high content of brittle minerals and overpressure in the formation.

##  Introduction

Shale gas is currently one of the most realistic replacement energy sources, the successful exploration of shale oil and gas in North America demonstrates the essential role of shale oil in the energy supply^1–3^. Research on shale micropores is currently the core content and frontier field of international shale gas research^4^. Loucks et al.^5^ recognised that micropores in shale are the main part of pores when defining the concept of “nano-pores”. Free and adsorbed gases in the micropores of shale are the basic hosts of shale gas^6–8^.

Unconventional shale oil and gas accumulations integrate sources, reservoirs, and caprocks, and shale oil and gas reservoirs may be preserved in specific places in the Yining Basin^9,10^. The oil and gas exploration began in the 1950s in the Yining Basin, however, no industrial oil and gas flows have yet been found. Some oil and gas fluorescence displays were found in the Permian shales of the Tiemulike Formation (P_2_t) in the Yican 1 and Yi 2 wells. The Permian oil and gas shows are very active on the land surface and are mainly oil-immersed sandstone, asphalt, asphalt veins, cave asphalt, and natural gas^11^. The oil and gas indicate that the Permian system in the Ili region underwent oil and gas generation, migration, accumulation, and post-reservoir destruction.

Previous shale studies have been conducted in the Yining Basin. Zhong^12^, Feng^11^, and Song^13^ have conducted extensive research on the sedimentary characteristics of the Tiemulike Formation; Feng et al.^14^ conducted a systematic study on the sedimentary environment of the black shale in the Tiemulike Formation. Miao et al.^15^, Xiong et al.^16^, Xie et al.^17^, and Feng et al.^11^ conducted a detailed study on the characteristics of hydrocarbon source rock of dark shale in the Tiemulike Formation. However, studies on the characteristics of shale micropores and their formation factors in the Tiemulike Formation of the Yining Basin are still scarce, which is one of the technical bottlenecks for shale oil and gas exploration in the Ili area.

The previous studies on the Yining Basin did not systematically address the shale micropore characteristics. Based on the scanning electron microscope analysis, microscopic observation, and whole-rock analysis of the Permian Tiemulike Formation, black shale samples from outcrop profiles and drilling cores in the Ili area, combined with the source rock characteristics of dark shales and the types and morphologies of micropores in the shales of the Tiemulike Formation, were analysed and described, and the main factors controlling the development of micropores were discussed. This study provides a basis for unconventional shale oil and gas exploration in the Yining Basin.

## Regional geological condition

### Geological setting

**Fig. 1:**
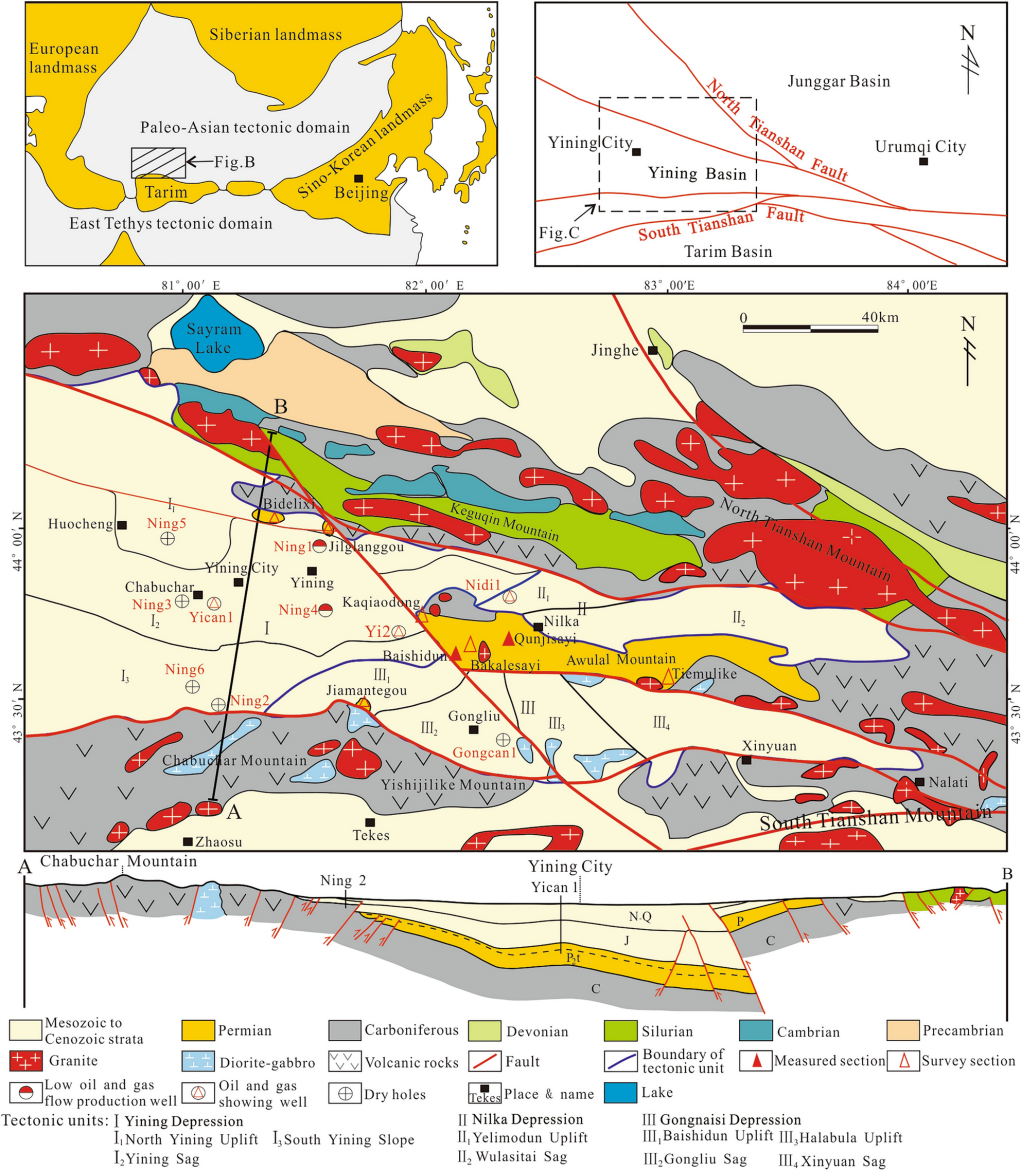
Location of the study area and the distribution of the geological overview of Yining basin (revised after^10,14,18,19^) (this figure is generated in CorelDRAW 2018 software, https://www.coreldrawchina.com/).

The Yining Basin is located in the Central Asian metallogenic domain^18^, and is a typical intermountain basin in the West Tianshan fold belt^19^ (Fig. 1A). The Yining Basin presents a “triangle” shape, the basin gradually shrinks and narrows in the North to South direction in the East (Fig. 1B), and the direction of most faults is consistent with the regional tectonic line (Fig. 1C).

**Fig. 2:**
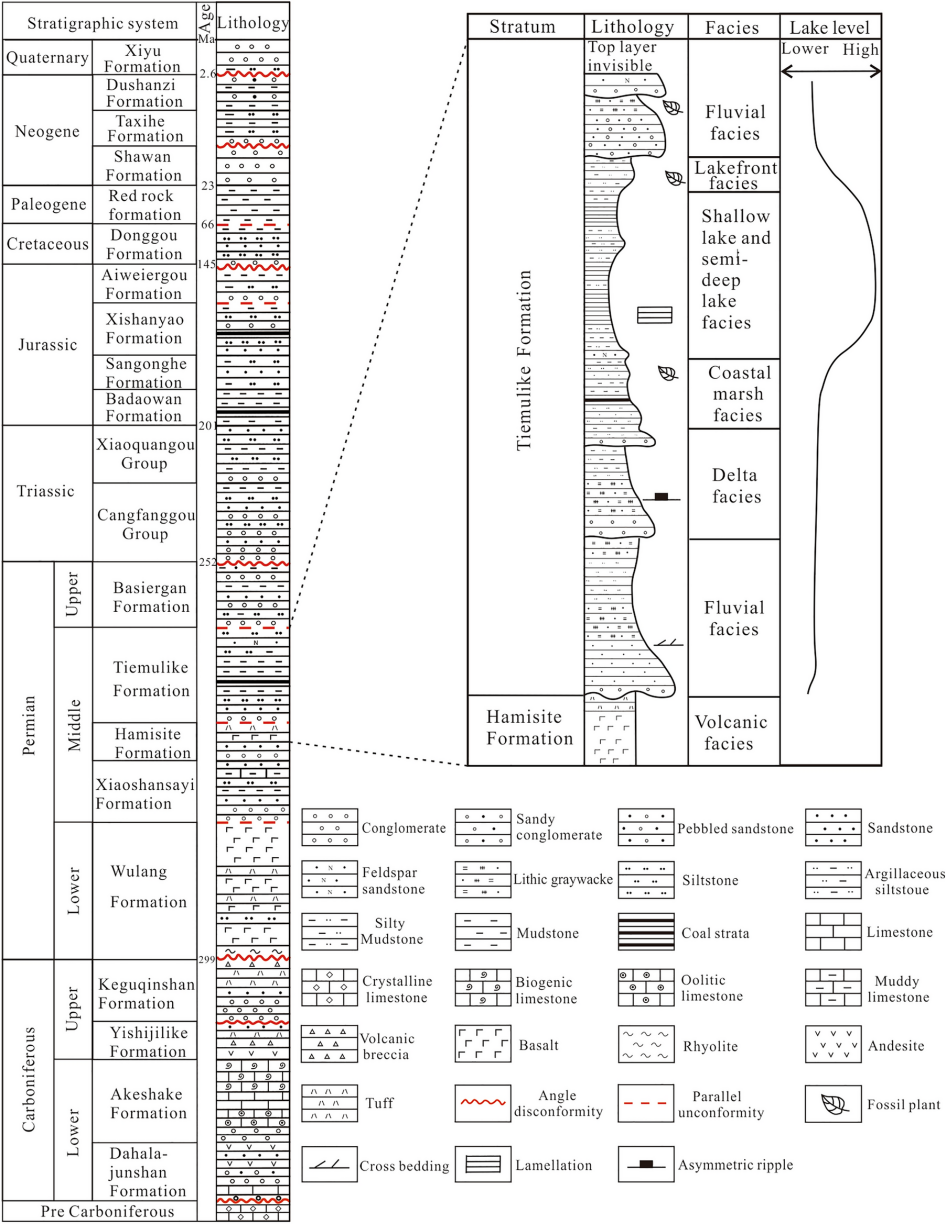
The stratigraphic column chart of the Yining Basin and the column chart of the Tiemulike Formation (this figure is generated in CorelDRAW 2018 software, https://www.coreldrawchina.com/).

There were four important evolutionary stages: the Neoproterozoic basement, the supercontinent breakup, the Post orogenic intraplate extensional, and the intracontinental^20–22^. The intracontinental stage can be divided into the intracontinental rift basin in the Permian and the intracontinental depression basins from the Triassic to the Cenozoic^23,24^. There were four strata series in Yining Basin, Neoproterozoic basement rock series, Carboniferous marine rift rock series, Permian intracontinental rift rock series, and Mesozoic intracontinental depression basin sedimentation to Cenozoic intermountain molasse accumulation (Fig. 2).

### Structural unit

There are three primary structural units in the Yining Basin, namely the Yining Depression in the West, Nilka Depression in the Northeast, and Gongnaisi Depression in the Southeast^25,26^. The shape of Yining Depression is a ladder. The Nilka Depression is a fault depression with an area of 2734 km^2^, and the Gongnaisi Depression is a fault depression with an area of 3600 km^2^. The Awulal Mountains are surrounded by the three depressions (Fig. 1C).

## Samples and methods

### Samples

This study collected 25 dark shale samples from the Tiemulike Formation, including samples from field outcrops and drilling core rocks such as the Qunjisayi, Bakalesayi, Baishidun, Kaqiaodong and Bidelixi sections and the Yican 1, Yi 2, Ning 1, Ning 3, and Ning 4 wells in Yining Basin. The sample collection task was systematically arranged for each sample point, and fresh samples were collected.

The instrument used for thin section identification was a Leica polarizing microscope, the model was Zeiss Axioskop 40. Scanning electron microscopy (SEM) and X-ray energy dispersive spectrometry (EDS) were used for microscopic morphology observation. The SEM model was JSM-7500F and the EDS model was X-Max 50. The magnification ranged from 25 to 1 × 10^6^, the resolution was 1.0 nm at 15 kV and 1.4 nm at 1 kV, and the acceleration voltage was 0.1 –30 kV. The instrument used for measuring the vitrinite reflectance (Ro) of kerogen in shale is a microscope photometer, the model was MPV-3, the testing environment is temperature 25 °C, relative humidity (RH) 50%. The Organic matter abundance, types and maturity were tested using the instrument of rock–eval pyrolysis, working voltage range 220 ± 10 v, working temperature range 10–30 °C, relative humidity less than 80%. An Axios X-ray fluorescence spectrometer was used for whole-rock analysis with the following conditions: the RSD was 0.1–1.0%, the SC was 1500 kcps, the FPC was 3000 kcps, and the test environment was 24 °C with 10 °C humidity. An Axioskop 40 polarisation electron microscope (Zeiss, Oberkochen, Germany; eyepiece: PL 10 × /23, object lens: A-PLAN, ACHROPLAN, PLAN, 4030 W halogen lamp, light source: HB050 and HB0103) was used for thin-section observation. The work of thin sections grinding was carried out in the laboratory of the regional geological and mineral research institute of the Shaanxi provincial bureau of geology and mineral exploration and development. Thin section identification, probe slices analysis, SEM observation, and X-ray diffraction (XRD) analysis were conducted at the test center of Xi’an Geological Survey Center, China Geological Survey. The test of shale geochemical characteristics were conducted at Oil and Gas Geochemistry Laboratory of Yangtze University.

### Methods

**Fig. 3:**
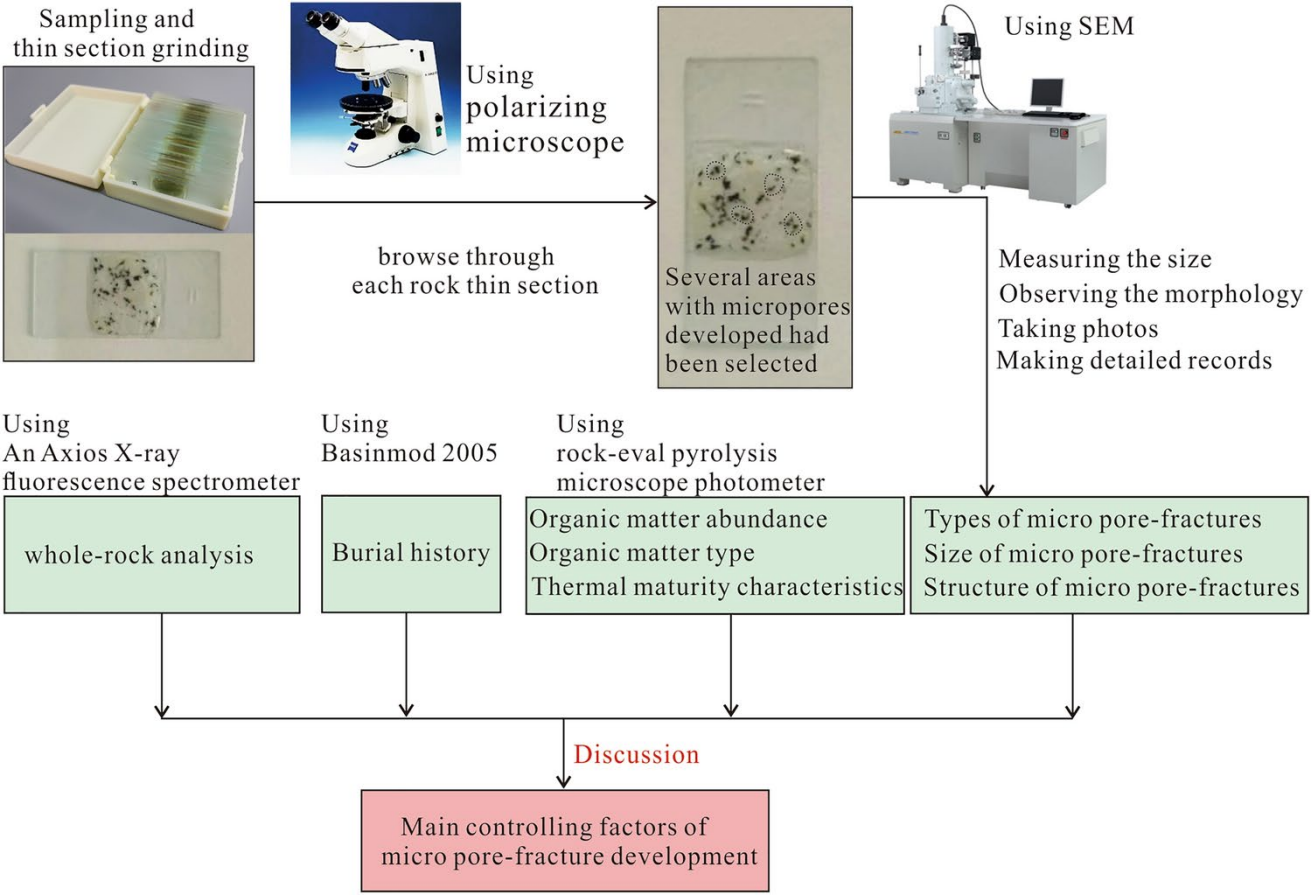
The flow chart for this study work (this figure is generated in CorelDRAW 2018 software, https://www.coreldrawchina.com/).

The main experimental work involved observing the micromorphology of the rocks. First, several areas with developed micropores were selected using a polarisation electron microscope to browse through each rock thin section. Then, by adjusting the microscope to the appropriate magnification, the morphology of micropores was closely observed, the size of micropores was measured, photos were taken, and detailed records were made. EDS was used to determine the composition of the minerals in the shale rocks. Approximately seven to nine points were observed for each shale sample. Third, the types of micropores were analysed. Finally, the whole-rock analysis and rock–eval pyrolysis were conducted, the geochemical characteristics of shale in Tiemulike Formation were performed. Combining the stratigraphic data, geochemical parameters of dark mud shale and so on of Ning 4 well, using the basin simulation software Basinmod, the burial history of Yining Basin was restored, and the main factors controlling micropore development were discussed (Fig. 3).

## Results

### Characteristics of shale

#### Distribution

The distribution of shale in the Tiemulike Formation was determined using method that combines geological outcrop surveys, drilling core observations, and two-dimensional seismic profile interpretation. Outcrops of the Tiemulike Formation are mainly distributed in the northern margin of the Yining Depression and around Awulela Mountain. The Tiemulike Formation in the Yining, Nilka, and Gongnaisi Depressions is covered by the overlying Mesozoic Cenozoic.

**Fig. 4:**
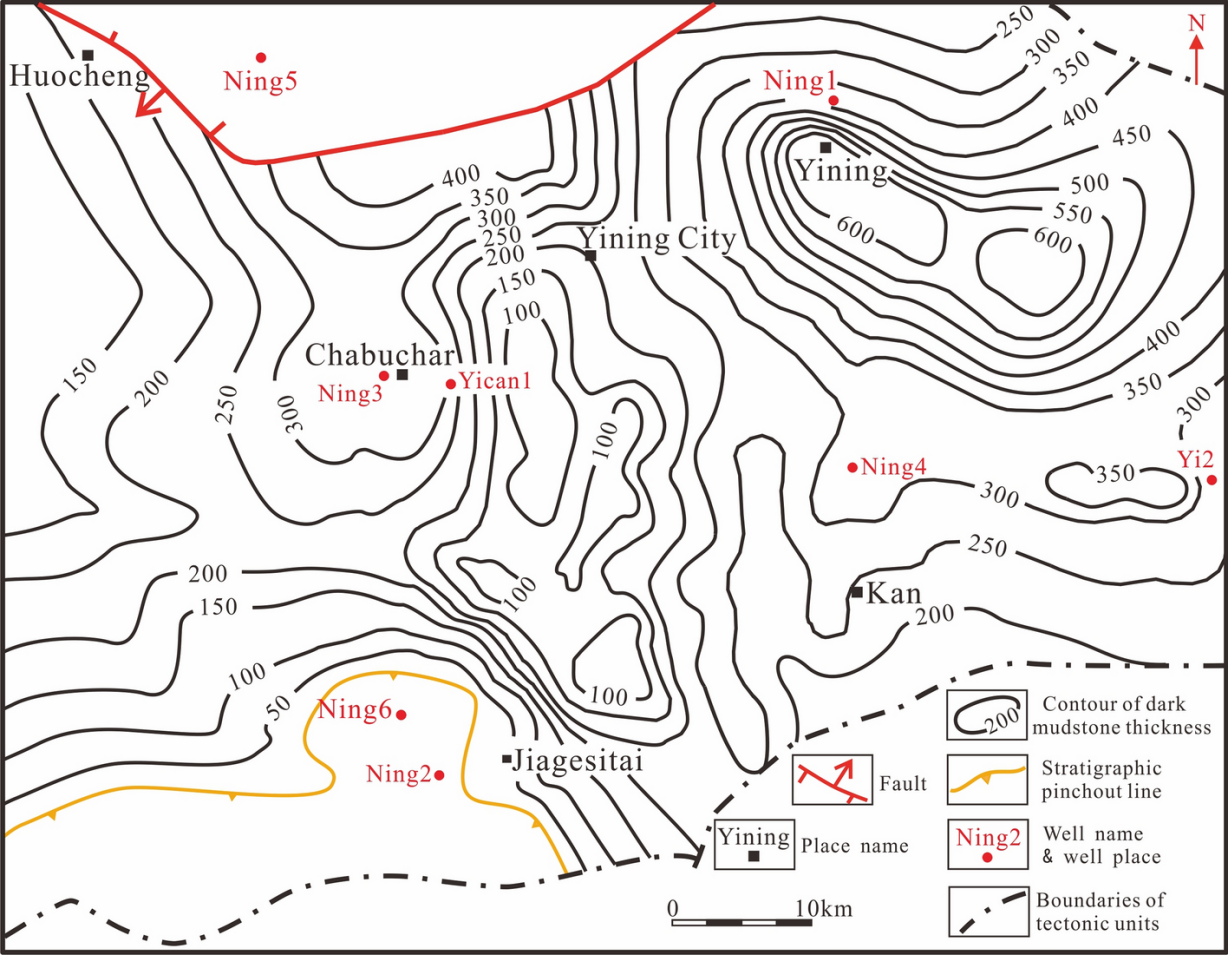
Contour map of dark mudstone thickness in the Tiemulike Formation (modified after 12) (this figure is generated in CorelDRAW 2018 software, https://www.coreldrawchina.com/).

The thickness of the dark mudstone in the Tiemulike Formation is mostly 150–600 m, and it is mostly 300–600 m in the area from North of Yining City to the Ning 4 well. The Tiemulike Formation was not deposited on the south-eastern edge of the Yining Depression. Under the control of tectonic movement from the Mesozoic to Cenozoic, the middle area east of the basin uplifted and Awulal Mountain formed. The residual thickness of the Tiemulike Formation gradually decreases from the edge of the depression to Awulal Mountain. The thicknesses of dark mudstone in the Tiemulike Formation are approximately 280 m, 130 m, and 150 m in the Yi 2 well, the Balekasayi section, and the Qunjisayi section, respectively (Fig. 4).

### Petrological characteristics

**Fig. 5:**
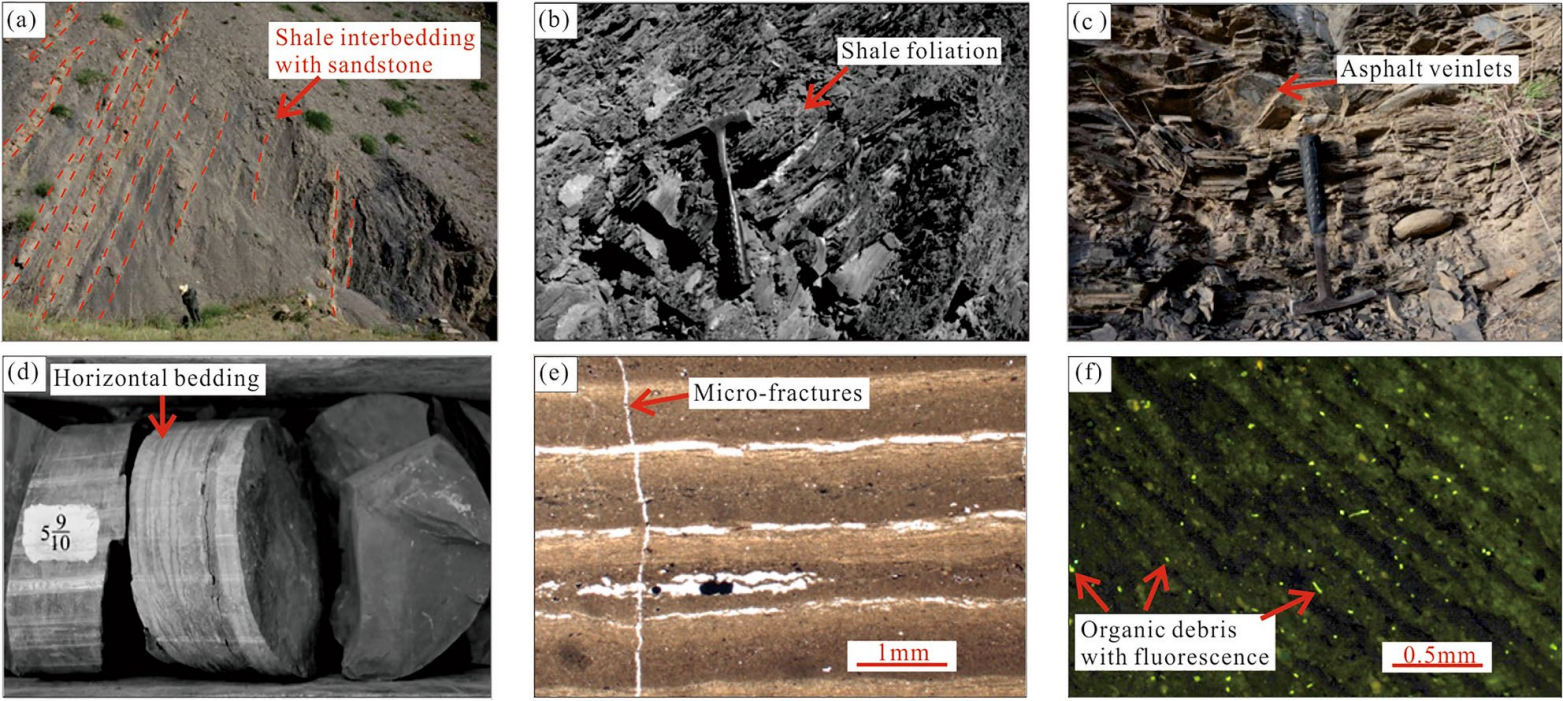
Characteristics of shale in Tiemulike Formation, Yining Basin. **a**Thick black shale intercalated with thin layer of grayish white siltstone in Qunjisayi section, **b** Black shale in Qunjisayi section, rich in organic debris and shale foliation developed, **c** Black shale intercalated with ellipsoidal siltstone lens in Qunjisayi section, asphalt veinlets are visible, **d** Black shale in Ning 3 well(4 297.1–4 299.1 m), Horizontal beddings developed, **e** Vertical micro-fractures and large number of micropores are visible in shales in Bakalesayi section, ×100(−), **f** Microscopic fluorescence photos of shale in Bakalesayi section, organic debris distributed as stars, ×100(−).

The area of paleo- Ili Lake expanded rapidly in the Late Guadalupian and the sediments of the Tiemulike Formation changed from coarse to fine. In the late sedimentary period of the Tiemulike Formation, the accommodation space of the lake basin became smaller, the water depth became shallower, and the sediment displayed a fine-to-coarse sequence vertically. According to observations of core rocks in the Yican 1, Yichan 2, Ning 3, and Ning 4 wells, and field outcrop rocks in the Qunjisayi, Bakalesayi, Baishidun, and Bidelixi sections of the Ili area, the Tiemulike Formation (P_2_t) mainly comprises black to dark-gray calcareous shale, mudstone, marl, gray sandstone, and fine conglomerate. The rock is relatively brittle and is strongly broken by weathering at the outcrops. Numerous joints and cracks developed in thick argillaceous rocks. Horizontal and wave bedding developed in the thick argillaceous rocks. Owing to seasonal changes, the colour of the rock stratum is rhythmic. Microscopic observations showed that the shale in the Tiemulike Formation is highly heterogeneous, with obvious light bands and dark bands rich in organic matter. Under ultraviolet rays, bands rich in organic matter exhibited blue-white and light-yellow fluorescence (Fig. 5). The sporopollen assemblage is characterised by Cordaitina-Crucisaccites-Protohap-Loxypinus, the pollen content of gymnosperms is 80–90%, the spores of ferns are rare, and the bivalve fossil assemblage is Palaeondonta-Palaeomutella-Neamnigenia*.* There was brackish water with high ancient salinity, alkalinity, and strongly reducing and anoxic environments. The sedimentary facies of the Tiemulike Formation are distributed in a nearly East–West direction on the plane, and comprise deep lake, shallow lake semi-deep lake, shore lake, flood plain, and fan delta facies.

### Mineral composition

**Fig. 6:**
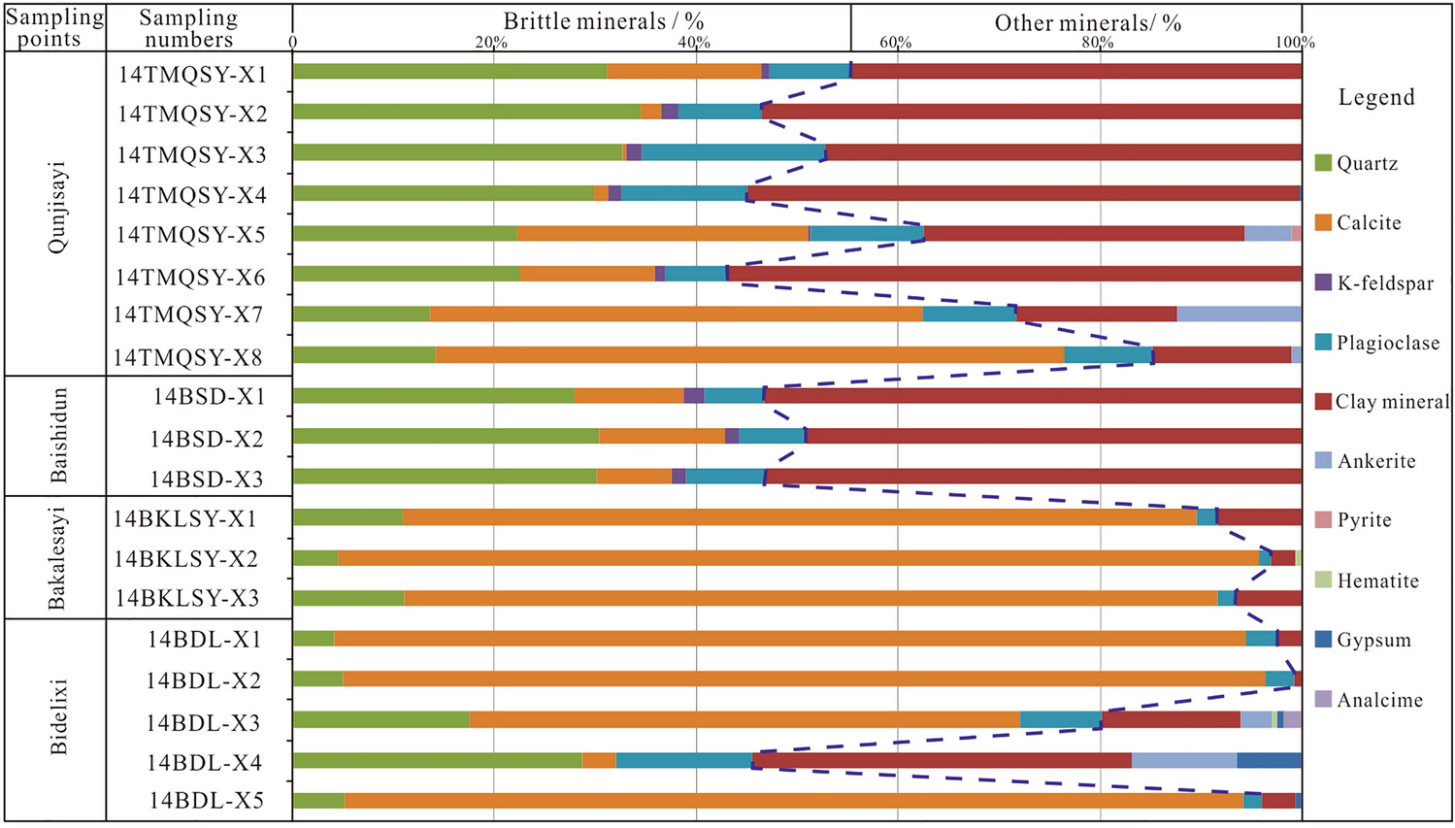
Mineral composition of whole rock of the shale in Tiemulike Formation (this figure is generated in CorelDRAW 2018 software, https://www.coreldrawchina.com/).

Shale in the Tiemulike Formation mainly comprises calcite, quartz, feldspar, and clay minerals in the Yining Basin. The calcite, quartz, and plagioclase contents ranged from 0.4 to 91.4%, 4.1 to 34.5%, and 1.1 to 18.2%, with averages of 41.2%, 19.8%, and 7.2%, respectively. The potassium feldspar (K-feldspar) content was approximately 1.0%. The clay mineral content ranged from 0.8 to 56.9% with an average of 28.9%. The brittle minerals in the shale of the Tiemulike Formation were mainly calcite, quartz, plagioclase, and K-feldspar, and the total brittle mineral content ranged from 45.1 to 99.2%, with an average of 68.8%. According to the data from eight samples from the Qunjisayi section of Awulal Mountain, the brittle mineral content ranged from 43.1 to 85.2%, with an average of 57.8%. According to the data from three samples from the Bashidun section of Awulal Mountain, the brittle mineral content ranged from 46.7 to 50.9%, with an average of 48.2%. Owing to the high calcite content, the brittle mineral content in the Bakarsayi section ranged from 91.6 to 96.9%, with an average of 94.0%. The brittle mineral content in the Bedlixi section ranged from 45.5 to 99.2%, with an average of 83.7%, and calcite accounted for approximately 90% of the total brittle mineral content (Fig. 6).

### Micro pores-fractures

The shale reservoir space in the Tiemulike Formation mainly comprises four types of micro pore-fractures in the Yining Basin. The micropores are mainly secondary pores, including three types of micropores: organic hydrocarbon-generating micropores, granular dissolved micropores, intergranular micropores, and the micropores formed by the hydrocarbon generation of organic matter is dominant.

#### Organic hydrocarbon-generating micro pores

**Fig. 7:**
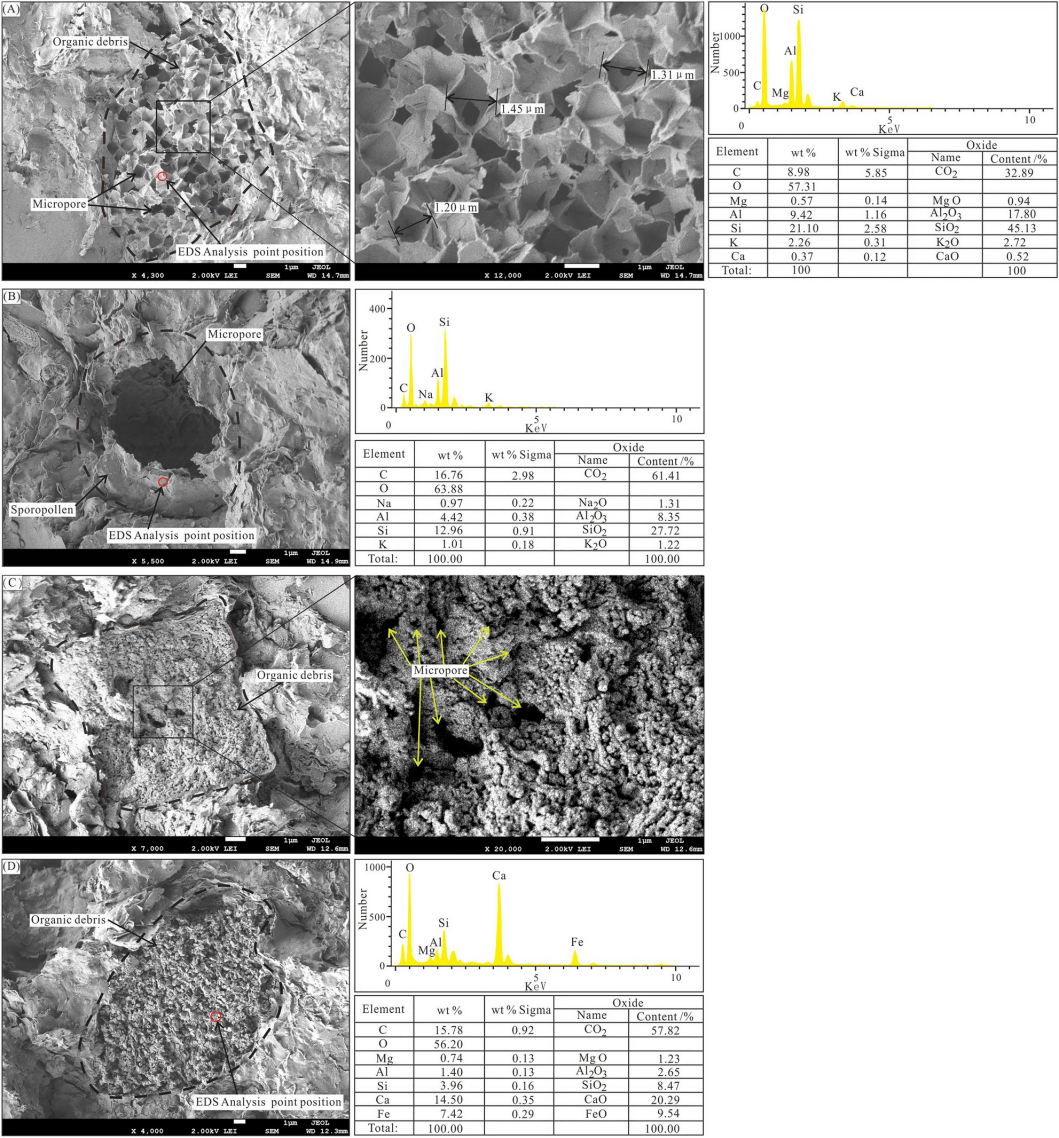
**A** Honeycomb shaped micropores in organic matter of shale in Tiemulike Formation in Qunjisayi section; **B** micropores formed due to the breakup of sporopollens in Qunjisayi section; **C** Dense micropores in organic matter in Baishidun section; **D** Dense micropores in organic matter in Qunjisayi section.

Certain micropores are formed by the hydrocarbon generation of organic matter. The dark shales in the Tiemulike Formation are rich in organic matter. Owing to the consumption of organic matter, many micropores were produced during hydrocarbon generation and evolution. The actual storage space of shale increases substantially during this process, which can improve its gas storage capacity. Jarvie and Lundell^27^ proved that carbonaceous residues are rich in micropores that arise from the thermal evolution of kerogen in organic shales. Researchers worldwide, such as Loucks et al.^4^, Ma^28^, and Feng^11^, have conducted much work in this regard. They found that micropores are formed through the thermal evolution of organic matter in shale, supporting the experimental results of Jarvie and Lundell^27^. The total organic carbon (TOC) content of dark shale in the Tiemulike Formation is 0.73–4.79% in the Yining Basin, with an average of 1.86%. The Ro at the edge of Yining basin is around 0.8%, while in the middle of the basin is mostly between 1.0 to 1.5%, with an average of 1.29%, and the thermal evolution degree is in the mature stage. Based on the SEM analysis, many micropores were observed, which were related to organic matter in the shales of the Tiemulike Formation. Owing to the hydrocarbon generation process of organic matter, the pores in the shale were mainly micropores, which formed owing to volume reduction. For honeycomb-like micropores, which developed in some organic matter, the size of a single micropore was approximately 1.20–1.45 μm (Fig. 7A). Owing to the breakup of pores, micropores formed during hydrocarbon generation in the dark shale (Fig. 7B). There were also some densely distributed micropores in the organic matter of shale, the size of which generally ranged from 10 to 100 nm (Fig. 7C, D).

#### Granular dissolved micro pores

**Fig. 8:**
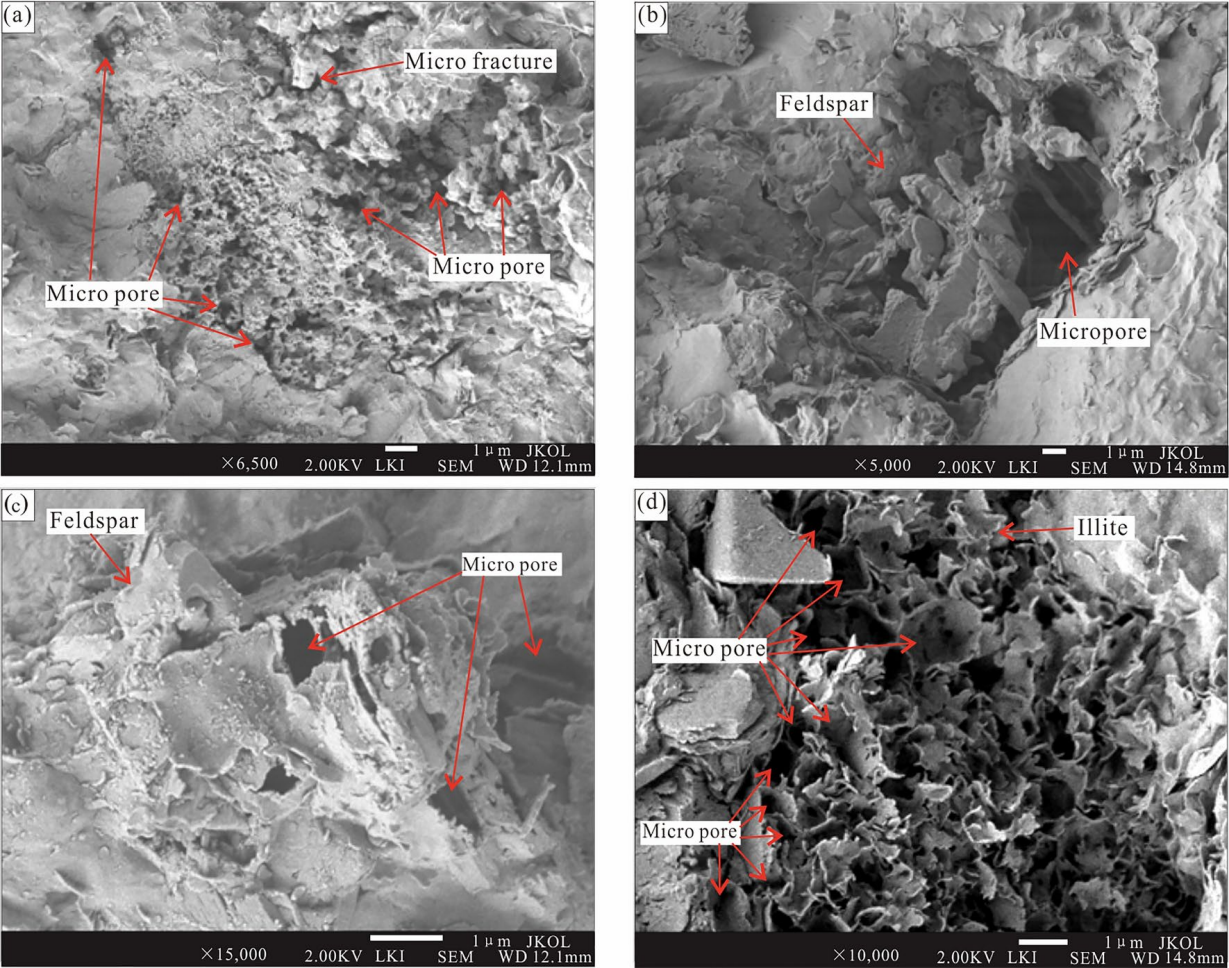
Micropores are formed during diagenesis of Shales in Tiemulike Formation at Qunjisayi section. **a** Large number of speckled micropores developed due to the dissolution of carbonate minerals, **b** Feldspar particles had been dissolved, and the residual skeleton became microporous, **c** Feldspar particles partially dissolved, causing lots of micropores, **d** the curly flaky Illites with numerous micropores.

Another type of micropore is dissolved micropores, which are formed by the dissolution of particles in shale. The SEM analysis showed that carbonate minerals were dissolved (Fig. 8A). Feldspar is unstable and easily dissolves during diagenesis, forming numerous dissolved micropores (Fig. 8B, C). According to the whole-rock composition of the dark shales in the Tiemulike Formation of the Yining Basin, the feldspar and calcite contents were high. The calcite content in some shale samples was approximately 90%. Micropores were easily produced during diagenesis. Due to the dehydroxylation of organic matter, the diagenetic environment became acidified in the middle diagenetic stage, carbonate minerals dissolved, and micropores were formed.

#### Intergranular micro pores

The third type of micropore is intergranular micropores, which are formed during the transformation of clay minerals in the Yining Basin. There were many clay minerals in the shales of the Tiemulike Formation, with an average content of 28.9%. During the process of burial and diagenesis, with the strengthening of diagenesis, the kaolinite and montmorillonite contents decreased, whereas those of chlorite and illite increased; curly flaky illite was common in the shales of the Tiemulike Formation, and there were many micropores between the illite flakes (Fig. 8D). The most important diagenetic change in the shale was the transformation of montmorillonite into illite, and many intergranular micropores developed.

### Micro-fractures

**Fig. 9:**
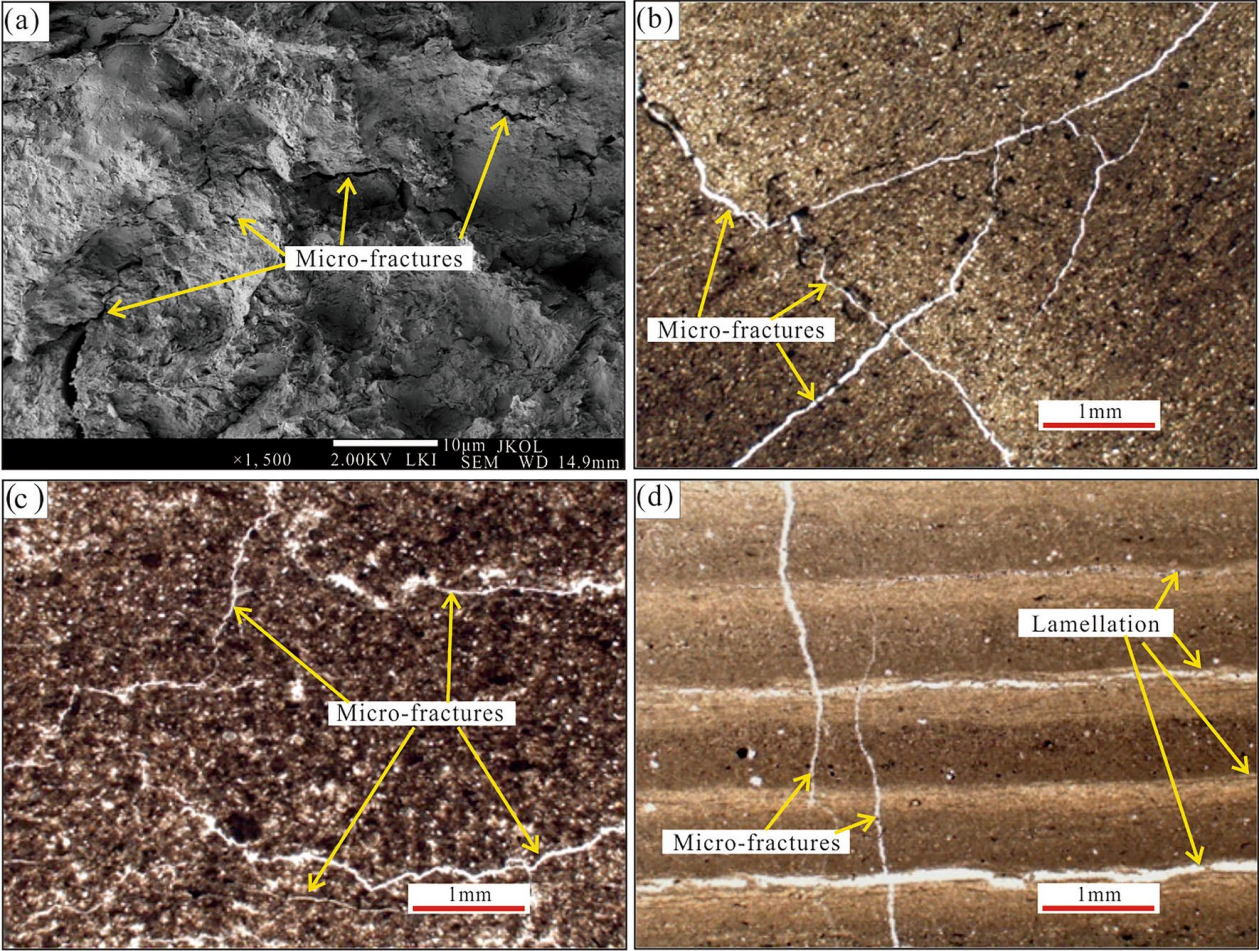
Photomicrograph of micro-fractures in shale of Tiemulike Formation at Qunjisayi section. **a** Morphology of micro-fracture observed by SEM, **b** interconnected network micro-fractures, ×100(−), **c** branched micro-fractures, ×100(−), **d** micro-fractures perpendicular to shale layer, ×100(−).

According to observations under a polarisation microscope and SEM, multiple micro-fractures can be observed in the shales of Tiemulike Formation in the Yining Basin. There were also micropores near the micro-fractures (Fig. 9a), which were potentially generated by dissolution. The extension distance of the micro-fractures was not far, and interconnections were observed between the micro-fractures. The end of one micro-fracture branched into a divergent shape and the surrounding micropores were connected (Fig. 9b, c). There was one type of micro-fracture perpendicular to the shale layer (Fig. 9D) in the shale of Tiemulike Formation. Many micro-fractures have developed in the shale of Tiemulike Formation, which can improve the physical properties of the reservoirs, thereby improving the adsorption capacity of shale gas.

## Discussion

### The influence of shale geochemical characteristics on micropore development

#### Organic matter abundance

**Fig. 10:**
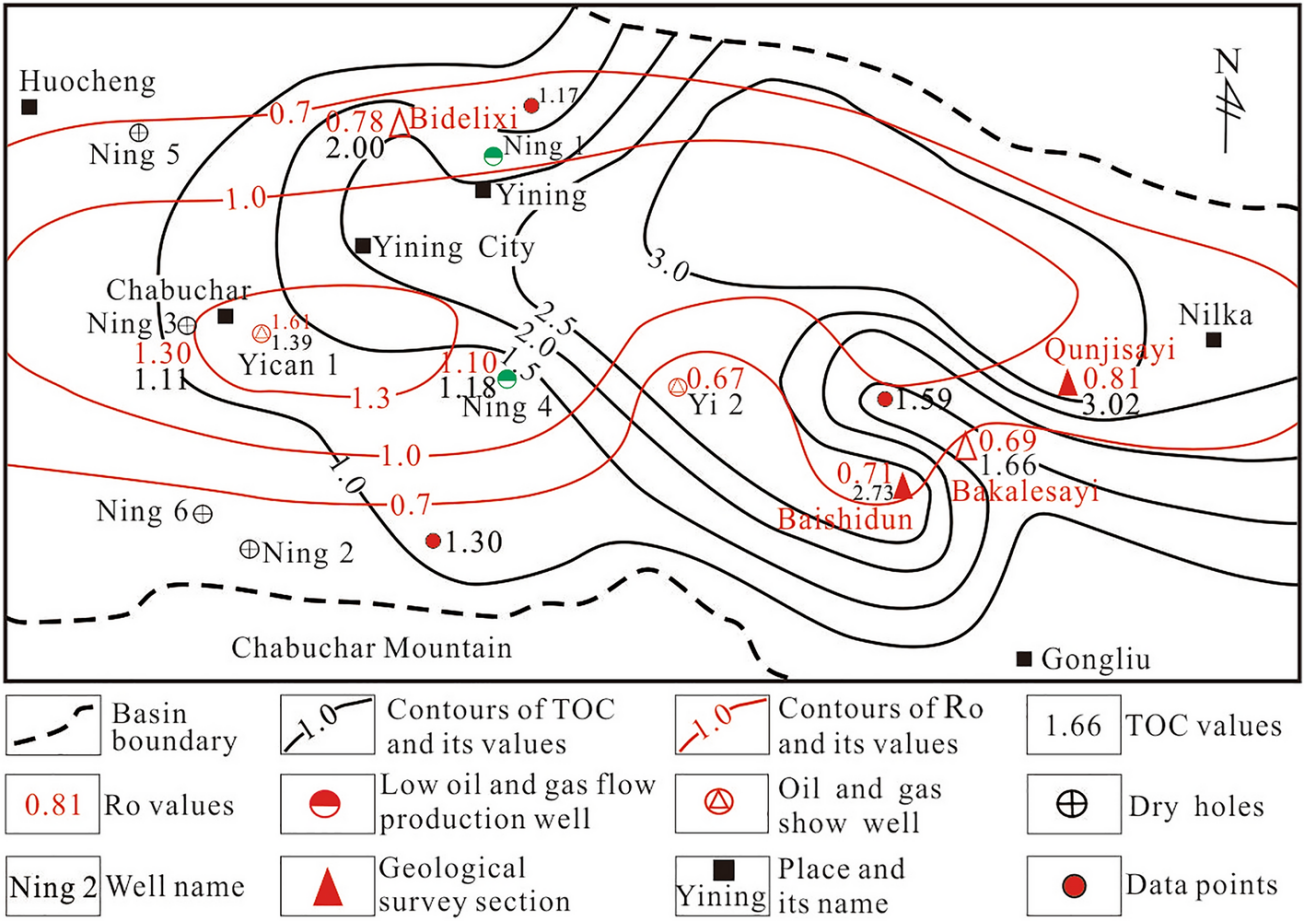
Superimposed distribution of TOC with Ro of the mud shale in Tiemulike Formation (this figure is generated in CorelDRAW 2018 software, https://www.coreldrawchina.com/).

The results of sample testing form this survey and collected previous test results show that the organic carbon (TOC) content of 522 samples ranges from 0.73 to 4.79%, with an average of 1.86%, and the abundance of TOC in the black shale tended to increase from the South to the North of the Yining Basin. The hydrocarbon generation potential (S_1_ + S_2_) of 240 samples ranges from 0.06 to 7.56 mg/g, with an average of 1.79 mg/g. The values of chloroform asphalt “A” from 86 samples ranged from 0.01 to 0.17%, with an average of 0.06%. The total hydrocarbon content (HC) values of 66 samples ranged from 11 to 1578 ppm, with an average of 467 ppm.The average TOC value was approximately 2.0%, and the average hydrocarbon generation potential value was approximately 3.5 mg/g from Yining to Nilka in the North area. The average TOC value was approximately 1.8%, and the average hydrocarbon generation potential value was approximately 0.8 mg/g from Ning 3, Yican 1, and Ning 4 to Bakalesayi area in the central area. The average TOC value was approximately 1.3%, and the average hydrocarbon generation potential value was approximately 0.3 mg/g at the slope belt in the Southern edge of the Yining Basin. The thermal evolution of the dark mud shale in the Tiemulike Formation of the Jiamantegou in the Gongnaisi Depression was influenced by later magmatic alteration, so the sampling data cannot reflect the true situation of this region (Table 1; Fig. 10).

**Table 1:** Statistical table of organic matter abundance parameters of dark mud shale in the Tiemulike formation.

Structural units		Outcrops/Drilling well	Layers	Lithology	TOC / %				S_1_ + S_2_/mg/g				Chloroform asphalt “A”/%				HC/ppm				Comprehensive evaluation	Data sources
					Sample number	Max	Min	Average	Sample number	Max	Min	Average value	Sample number	Max	Min	Average	Sample number	Max	Min	Average		
Nilka depression		Qunjisayi	P_2_^t^	Black mud shale, Silty mudstone	41	4.91	0.17	3.02	41	12.54	0.02	4.91	12	0.55	0.00	0.17	12	265	15	173	High quality	This paper
		Qunjisayi	P_2_^t^	Grey black mud shale	5			2.58	5			2.80	2			0.14	1			595	Good	^16^
		Qunjisayi	P_2_^t^	Dark gray marl	28	7.21	0.25	2.14													High quality	^29^
				Grey black mud shale	163	10.80	0.15	2.13													High quality	
		Nilka	P_2_^t^	Grey black mud shale	13	4.35	0.14	1.11	13	5.91	0.04	0.71	6	0.15	0.01	0.04	6	652	20	236		^15^
Yining depression	East part	Bakalesayi	P_2_^t^	Black mud shale	7	2.71	1.04	1.66	7	0.60	0.09	0.30	3	0.01	0.01	0.01		350	96	187		This paper
		Bakalesayi	P_2_^t^	Dark gray marl	7	2.91	0.64	1.34	7	3.69	0.09	0.73	7	0.11	0.01	0.03	5	616	50	236	Good	
		Bakalesayi	P_2_^t^	Grey black mar	8	2.73	0.39	0.98													Good	^29^
				Black mud shale	6	1.77	0.43	1.15													Good	
		Bakalesayi	P_2_^t^	Grey black marl	58			1.32	56			2.01	18	_	_	0.14	13			700	Good	^30^
		Baishidun	P_2_^t^	Black silty mud shale	27	4.11	0.22	2.73	27	1.20	0.04	0.55	8	0.01	0.00	0.01	8	62	14	30	Good	This paper
		Kaqiaodong	P_2_^t^	Grey black mud shale	3			1.59	3			0.06	1			0.01	1			11	Medium	^30^
	West part	Yican1	P_2_^t^	Black mud shale and marl	7	1.84	0.34	0.73	7	0.49	0.03	0.18	7	0.06	0.01	0.02					Medium	^16^
		Yican1	P_2_^t^	Black mud shale and marl	30	4.79	0.35	1.39	29	8.29	0.05	0.98	8	_	_	0.03	5			225	Good	^30^
		Ning3	P_2_^t^	Grey mud shale	33	2.37	0.36	1.11	33	2.17	0.10	0.36	9	0.01	0.04	0.02	8	62	246	121	Medium	
		Ning4	P_2_^t^	Black mud shale	10	2.33	0.80	1.18	10	1.14	0.09	0.34	10	0.00	0.03	0.01					Medium	
	North part	Jilglanggou	P_2_^t^	Grey black mud shale	4			4.79	4			7.56	2			0.16	2			634	Good	^30^
		Jilglanggou	P_2_^t^	Dark gray marl	1			0.81	1			0.26	1			0.01					Poor	^16^
		Bidelixi	P_2_^t^	Grey black mud shale	12	4.34	0.59	2.54	12	26.12	0.89	8.24	9	0.57	0.03	0.37	9	2455	58	637		^15^
		Bidelixi	P_2_^t^	Black marl	14	3.86	0.07	1.50													High quality	^29^
				Black mud shale	60	5.90	0.08	2.60													Good	
		Bidelixi	P_2_^t^	Black mud shale	11	2.92	0.05	1.17	11	2.69	0.54	1.41	4	0.04	0.01	0.03	11	5837	161	1578		This paper
Gongnaisi depression		Jiamantegou	P_2_^t^	Grey black mud shale	5			1.41	5			0.50									Poor-non	31
		Jiamantegou	P_2_^t^	Dark gray mud shale	4	2.51	0.72	1.21	4	0.49	0.04	0.16	4	0.02	0.00	0.01					Poor-non	^30^

### Organic matter type

**Fig. 11:**
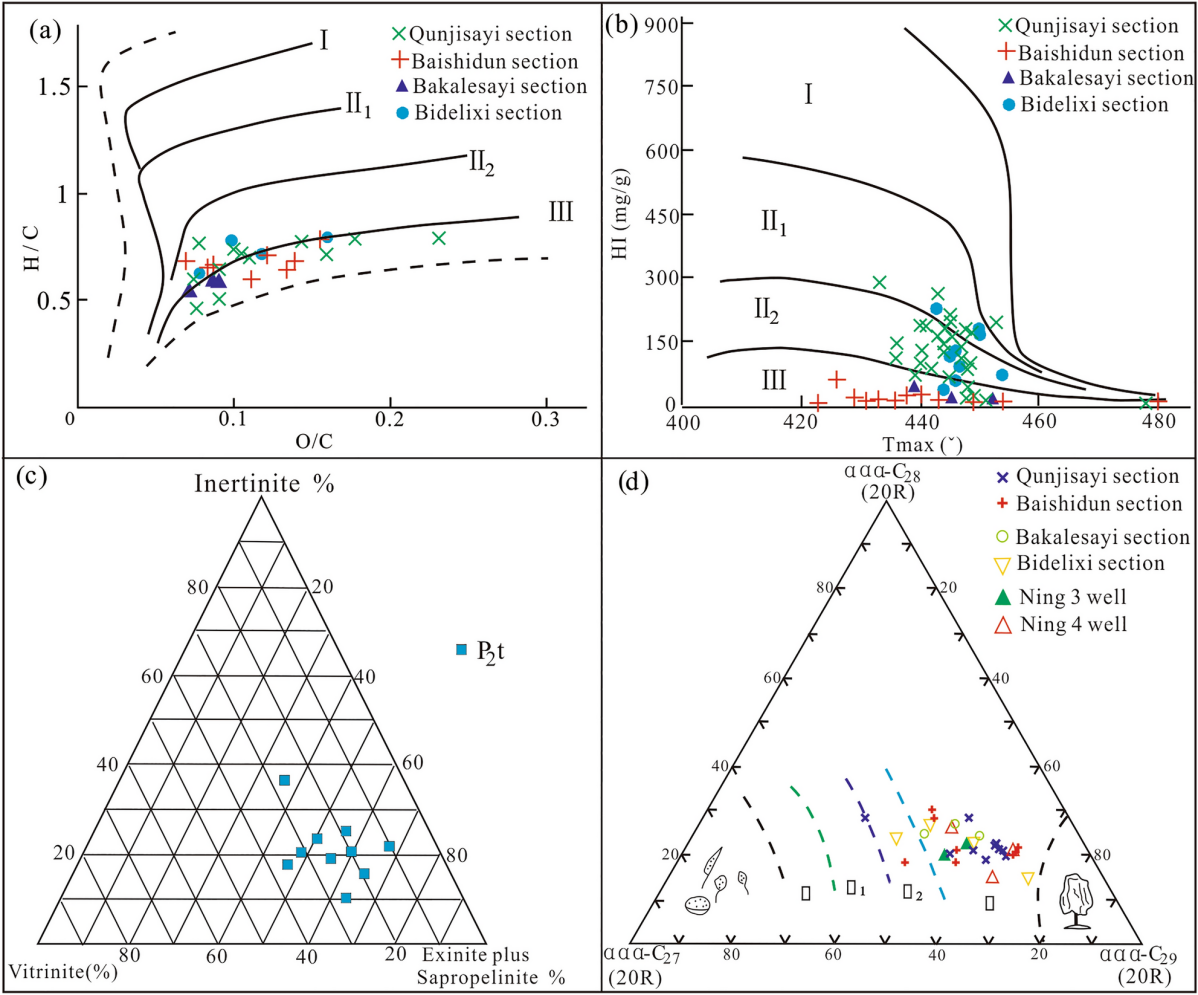
Diagram of organic matter type discrimination for dark mud shale in the Tiemulike formation. **a** Discriminant diagram of organic matter type using the correlation between H/C and O/C, **b** Discriminant diagram of organic matter type using the correlation between HI index with Tmax, **c** Microscopic components in the dark mudstone of the Tiemulike Formation in Well Ning 3, **d** Triangular diagram of organic matter type using the relative composition of Sterane (this figure is generated in CorelDRAW 2018 software, https://www.coreldrawchina.com/).

The type of kerogen in the mud shale of the Tiemulike Formation was mainly type III, with partly type II_2_ (Fig. 11). From the discriminant diagram of organic matter type using the correlation between H/C and O/C (Fig. 11a) and the discriminant diagram of organic matter type using the correlation between HI index with Tmax (Fig. 11b), It can be concluded that the main types of organic matter kerogen are type III-II_2_, with a small amount of type I and type II_1_. From the microscopic components in the dark mud shale of the Tiemulike Formation, take Ning3 well as an example (Fig. 11c), the content of sapropel and chitin is relatively high. The content range of sapropel group is 0–97.3%, mostly ranging from 10–70%, with an average of about 40%. The content range of chitin is 0–81%, mostly between 20–40%, with an average of 30%. The content of vitrinite is 6–87.5%, mostly 20–30%, with an average of about 25%. The content of inertinite is 0–62.5%, mostly 10–25%, with an average of about 15%. These data indicate that the mud shale of the Tiemulike Formation has strong hydrocarbon generation potential, and the kerogen types are classified into II_2_-III types. As we all known, the biomarker compounds are less affected by weathering. From the triangular diagram of organic matter type using the relative composition of sterane (Fig. 11d), the C_29_ sterane dominates in steranes, from which can distinguish that the organic matter type is mainly type III, with a small amount of type II_2_.

### Thermal maturity characteristics

In this paper, the maturity of the mud dark shale in the Tiemulike Formation was evaluated using indicators such as specular reflectance Ro and rock pyrolysis parameter Tmax, the discrimination standard is SY/T5735-1995. The distribution characteristics of reflectance of dark mudstone vitrinite are shown as in Fig. 10, the degree of thermal evolution of the dark shale in the Tiemulike Formation gradually increased from the edge to the central area of the Yining Basin. The value of Ro was approximately 0.8% at the edge, and the dark shale recently entered the mature stage in the source rock evolution process. The value of Ro mostly ranged from 1.0 to 1.5% in the central area. The weighted average value of Ro was 1.29%, and the dark shale entered a highly mature stage in the source rock evolution process (Fig. 10).

**Fig. 12:**
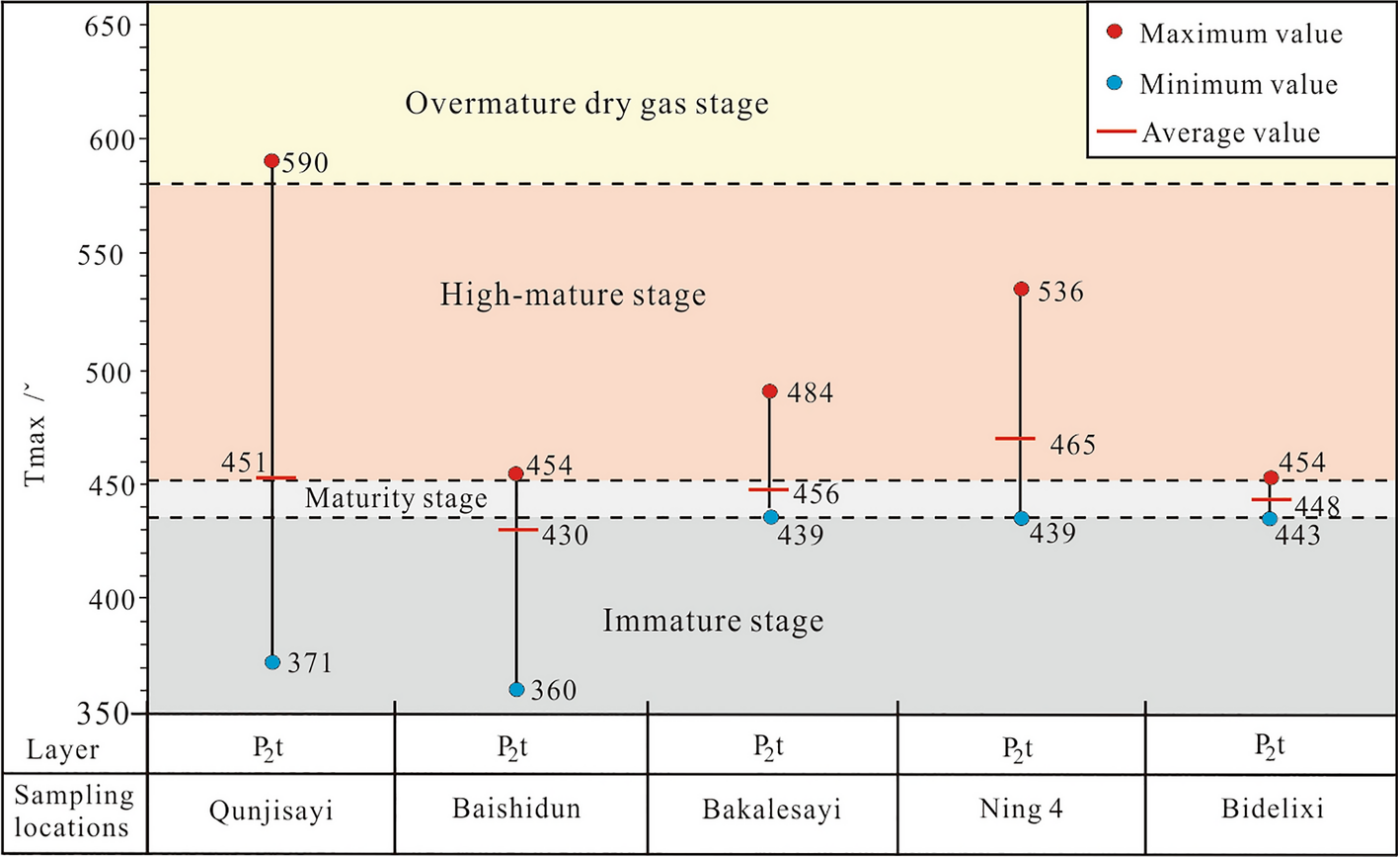
Distribution of pyrolysis temperature peaks of dark mud shale in the Tiemulike Formation (this figure is generated in CorelDRAW 2018 software, https://www.coreldrawchina.com/).

Based on the 86 sample testing data obtained from field geological profiles and 55 sample testing data collected from previous, the distribution of pyrolysis temperature peaks of dark mudstone in Tiemulike Formation of the Yining Basin has been drawn (Fig. 12). The results indicate that the dark mud shale of the Tiemulike Formation in the central Yining Depression and the Nilka Depression has mostly entered the high maturity stage, the dark mud shale of the Tiemulike Formation in the northern part of the Yining Depression is in the mature stage, the maturity of the dark mud shale evolution in the Tiemulike Formation in the eastern part of the Yining Depression is the lowest, and most of them are still in the low maturity stage.

### The burial history

**Fig. 13:**
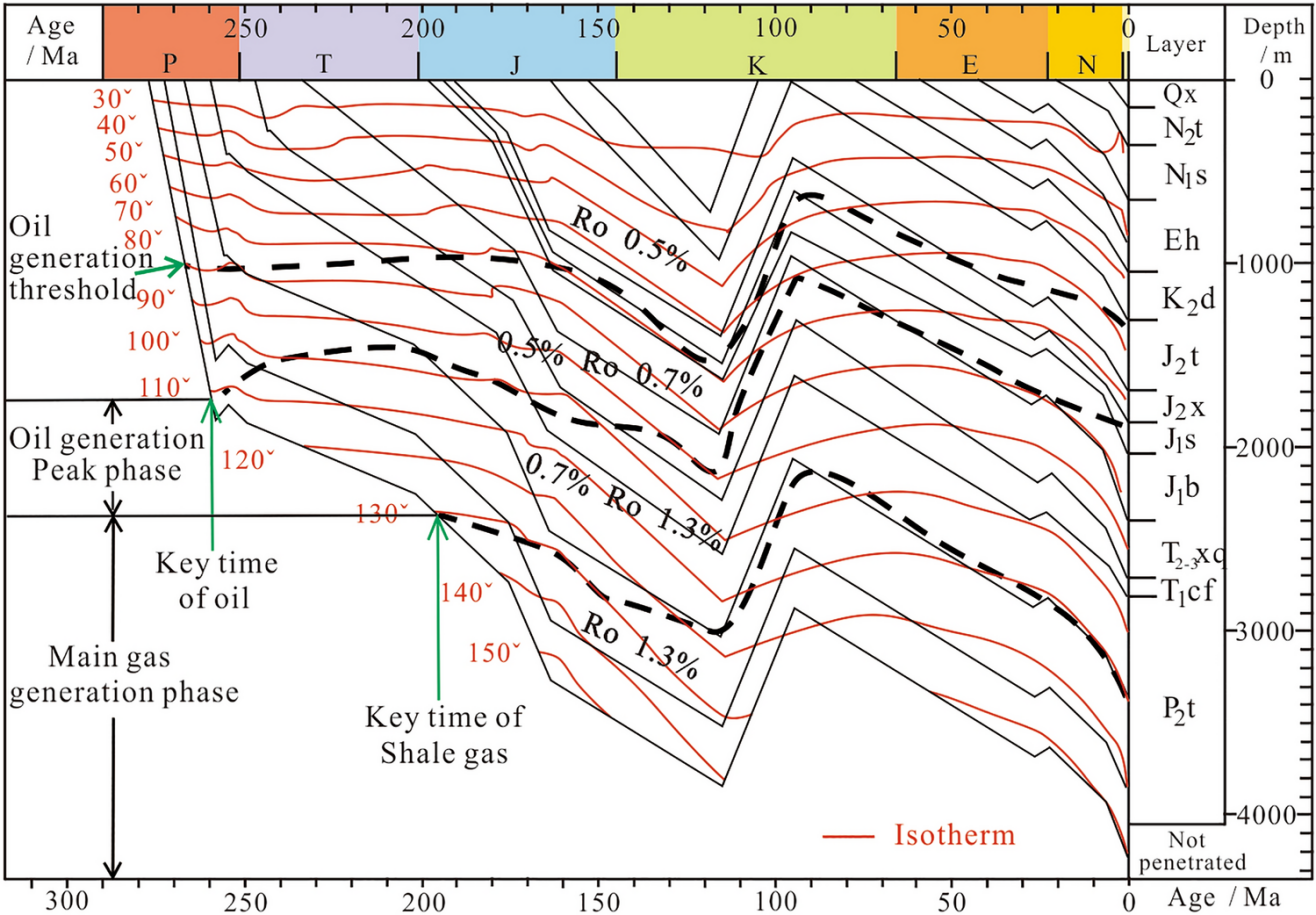
Burial history of Tiemulike Formation in Ning 4 well of the Yining basin (this figure is generated in CorelDRAW 2018 software, https://www.coreldrawchina.com/).

Combining the stratigraphic data, geochemical parameters of dark mud shale and so on of Ning 4 well (Table 2), using the basin simulation software Basinmod, the burial history of Yining Basin was restored. The research results indicate that Yining basin had experienced mainly four subsidence and erosion stages since Permian. The maximal sedimentation rate existed from middle Permain to early Triassic, and basin uplift in a short time in late Permain. While basin quickly deposited from early Triassic to late Cretaceous, what is more, Yining Basin had a large scale uplift and erosion in early Cretaceous, and sedimentation rate decreased from early stage of late Cretaceous to the end of Paleogene, in which basin quickly uplift caused by the effect from Himalayan movement. The basin slowly deposited from early Neogene to late Pleistocene, and it is in the erosion condition nowadays (Fig. 13).

**Table 2:** Basic data for reconstruction of burial history of Ning 4 in the Yining basin.

Formation or Event name	Tectonic type	Begin age/Ma	Top depth/m	Present thick/m	Eroded thickness/m	Lithology	Ro/%	Kerogen type/%
Present	Erision	0			180			
Q*x*	Sedimentation	1.21	0	150		Sandstone		
N_2_*t*	Sedimentation	2.59	150	208		Sandstone (7%) + Shale (93%)		
N_1_*s*	Sedimentation	5.33	358	298		Sandstone (48%) + Shale (34%) + Siltstone (18%)		
E*h*	Sedimentation	23.03	656	392.5		Limestone (2%) + Siltstone (11%) + Shale (87%)		
K_2_*d*	Sedimentation	66	1048.5	260.5		Sandstone (18%) + Shale (72%) + Siltstone (10%)		
J_3_-K_1_	Erision	95			989			
J_3_-K_1_	Formation	115						
J_2_*t*	Sedimentation	163.5	1309	381		Sandstone (15%) + Shale (65%) + Siltstone (20%)		
J_2_*x*	Sedimentation	168.3	1690	175.5		Sandstone (25%) + Shale (25%) + Siltstone (50%)	0.39(coal)	III
J_1_*s*	Sedimentation	174.1	1865.5	169		Sandstone (16%) + Shale (62%) + Siltstone (22%)	0.45(coal)	III
J_1_*b*	Sedimentation	182.7	2034.5	367.5		Sandstone (13%) + Shale (55%) + Siltstone (32%)		III
T2-3xq	Sedimentation	201.3	2402	308		Sandstone (6%) + Shale (81%) + Siltstone (13%)	0.62	II_2_
T_1_*cf*	Sedimentation	247.2	2710	95		Sandstone (63%) + Shale (16%) + Siltstone (21%)		
	Erision	251.2			60			
	Sedimentation ceases	252.2						
P_2_*t*	Sedimentation	259.9	2805	1242.34 (Not penetrated)		Sandstone (6%) + Shale (85%) + Siltstone (9%)	1.10	III-II_2_

### TOC content and thermal evolution controlled the development of micropores

Many micropores developed under the coupling effect of the high organic carbon content with a high degree of thermal evolution. The micropores formed during the thermal evolution of organic matter and hydrocarbon generation are the main pores in shale reservoirs of Tiemulike Formation. During the thermal evolution of hydrocarbon generation in the mud shale of the Tiemulike Formation, the volume of organic matter in dark shale had reduced, resulting in the formation of numerous micro pores. Due to the consumption of organic matter through hydrocarbon generation and evolution, a large amount of pore space has been increased, greatly increasing the actual storage space of shale. The superimposition area of high organic carbon content area and high vitrinite reflectance area is the main micropore developed area (Fig. 10).

The Ili block began to subside rapidly from the Late Permian, and the dark shale in the lower part of the Tiemulike Formation reached the hydrocarbon generation threshold from the end of the Late Permian to the early Early Triassic. Owing to rapid sedimentation, the dark shale was continuously heated, generating oil and gas. The dark shale reached its peak oil generation in the Early Triassic and entered the dry gas stage in the Late Jurassic. The burial history of the Yining Basin shows that the stratum of the Tiemulike Formation reached a maximum burial depth in the late Early Cretaceous, and the burial depth was > 4000 m. Therefore, the residual primary pores in shale of the Tiemulike Formation is a little or no. During continuous hydrocarbon generation, numerous micropores and micro-fractures formed in the dark shale of the Tiemulike Formation. Micropores formed during the thermal evolution of organic matter and hydrocarbon generation are the main pores in the shale reservoirs of the Tiemulike Formation. Micropores are mainly developed in areas of high TOC content with a high Ro value, the micropores formed by the hydrocarbon generation of organic matter is dominant.

### The influence of brittle minerals on the development of micropores

According to whole-rock analysis data, the high brittle mineral content is an internally favourable condition for the development of micro-fractures in the shale of the Tiemulike Formation (Fig. 6). Under the stress of geological tectonics, the micro-fractures formed easily because of the high brittle mineral content in the shale of the Tiemulike Formation. The cleavages in the shale of the Tiemulike Formation at geological outcrops indicate the existence of tectonic stress. By observing rock slices with a microscope, the natural micro-fractures were found to have developed in the shale of the Tiemulike Formation. The organic matter is also a ductile component, ductile clay also can generate fractures by rotation and slip of clay plates.

Simultaneously, the overpressure is closely related to the micro-fracture in the shale reservoir^20,32^. The type of kerogen in the mud shale of the Tiemulike Formation was mainly type III, with partly type II_2,_ which is conducive to generating a large amount of natural gas. During the thermal evolution of hydrocarbon generation in the shale of the Tiemulike Formation, a large quantity of hydrocarbon-bearing fluids is produced, which causes overpressure in the shale, thus forming a pressure compartment. When the excess pressure exceeds the compressive strength of the shale, the fluids in the pressure compartment break through the shale layers and flow out. Because of the high brittle mineral in the shale of the Tiemulike Formation, micro-fractures developed during the evolution of hydrocarbon generation, which occurred repeatedly in the dark shale; therefore, a many micro-fractures developed.

## Conclusion

The shale reservoir space in the Tiemulike Formation mainly comprises four types of micro pore-fractures in the Yining Basin. The micropores are mainly secondary pores, including three types of micropores, and the micropores formed by the hydrocarbon generation of organic matter is dominant. The first type is organic hydrocarbon-generating micropores, and the size of most single micropores is approximately 1.20 to 1.45 μm. The second type is granular dissolved micropores, mainly the dissolution of feldspar and calcite. The third type is intergranular micropores, which are formed during the transformation of clay minerals in the Yining Basin. Owing to the high content of brittle minerals, with an average of 68.8%, there were several micro-fractures. The extension distance of the micro-fractures was not far, with interconnections between the micro-fractures. The end of one microfracture branched into a divergent shape and the surrounding micropores were connected.

Many micropores developed under the coupling effect of a high organic carbon content with a high degree of thermal evolution, which is the main body of micropores in the shale of the Tiemulike Formation. A high brittle mineral content is an internally favourable condition for the development of micro-fractures in the shales of the Tiemulike Formation. Owing to the thermal evolution of shale organic matter, overpressure is essential in boosting the generation of numerous micro-fractures.

## Data Availability

The data are available from the corresponding author upon reasonable request.
